# Common Variants in 22 Genes Regulate Response to Metformin Intervention in Children with Obesity: A Pharmacogenetic Study of a Randomized Controlled Trial

**DOI:** 10.3390/jcm8091471

**Published:** 2019-09-16

**Authors:** Augusto Anguita-Ruiz, Belén Pastor-Villaescusa, Rosaura Leis, Gloria Bueno, Raúl Hoyos, Rocío Vázquez-Cobela, Miriam Latorre-Millán, M. Dolores Cañete, Javier Caballero-Villarraso, Ángel Gil, Ramón Cañete, Concepción M. Aguilera

**Affiliations:** 1 Department of Biochemistry and Molecular Biology II, Institute of Nutrition and Food Technology “José Mataix”, Center of Biomedical Research, University of Granada, Avda. del Conocimiento s/n. Armilla, 18016 Granada, Spain; augustoanguitaruiz@gmail.com (A.A.-R.); agil@ugr.es (Á.G.); caguiler@ugr.es (C.M.A.); 2 Instituto de Investigación Biosanitaria IBS.GRANADA, Complejo Hospitalario Universitario de Granada, 18014 Granada, Spain; 3 CIBEROBN (Physiopathology of Obesity and Nutrition Network CB12/03/30038), Institute of Health Carlos III (ISCIII), 28029 Madrid, Spain; mariarosaura.leis@usc.es (R.L.); gbuenoloz@yahoo.es (G.B.); 4 LMU – Ludwig-Maximilians-University of Munich, Division of Metabolic and Nutritional Medicine, Dr. von Hauner Children’s Hospital, University of Munich Medical Center, 80337 Munich, Germany; 5 Unit of Investigation in Nutrition, Growth and Human Development of Galicia, Pediatric Department, Clinic University Hospital of Santiago, University of Santiago de Compostela, 15706 Santiago de Compostela, Spain; cobela.rocio@gmail.com; 6 Pediatric Department, Lozano Blesa University Clinical Hospital, University of Zaragoza, 50009 Zaragoza, Spain; latorremiriam0@gmail.com; 7 Pediatric Department, Virgen de las Nieves University Hospital, Andalusian Health Service, 18014 Granada, Spain; raul_hoyosgurrea@yahoo.es; 8 Health Sciences Institute in Aragon, 50009 Zaragoza, Spain; 9 PAIDI CTS-329, Maimonides Institute of Biomedical Research of Córdoba (IMIBIC), 14004 Córdoba, Spain; mdcanete@hotmail.com (M.D.C.); em1caesr@uco.es (R.C.); 10 Clinical Analysis Services, IMIBIC/Reina Sofía Hospital, University of Córdoba, 14004 Córdoba, Spain; bc2cavij@uco.es; 11 Unit of Pediatric Endocrinology, Reina Sofia University Hospital, 14004 Córdoba, Spain

**Keywords:** metformin, obesity, pediatrics, SNP, pharmacogenetics, clinical trials

## Abstract

Metformin is a first-line oral antidiabetic agent that has shown additional effects in treating obesity and metabolic syndrome. Inter-individual variability in metformin response could be partially explained by the genetic component. Here, we aimed to test whether common genetic variants can predict the response to metformin intervention in obese children. The study was a multicenter and double-blind randomized controlled trial that was stratified according to sex and pubertal status in 160 children with obesity. Children were randomly assigned to receive either metformin (1g/d) or placebo for six months after meeting the defined inclusion criteria. We conducted a post hoc genotyping study in 124 individuals (59 placebo, 65 treated) comprising finally 231 genetic variants in candidate genes. We provide evidence for 28 common variants as promising pharmacogenetics regulators of metformin response in terms of a wide range of anthropometric and biochemical outcomes, including body mass index (BMI) Z-score, and glucose, lipid, and inflammatory traits. Although no association remained statistically significant after multiple-test correction, our findings support previously reported variants in metformin transporters or targets as well as identify novel and promising loci, such as the *ADYC3* and the *BDNF* genes, with plausible biological relation to the metformin’s action mechanism. Trial Registration: Registered on the European Clinical Trials Database (EudraCT, ID: 2010-023061-21) on 14 November 2011 (URL: https://www.clinicaltrialsregister.eu/ctr-search/trial/2010-023061-21/ES).

## 1. Introduction

The prevalence of overweight and obese children is a serious worldwide issue and one of the major health challenges of the 21st century [1]. Childhood obesity plays an important pathophysiologic role in the development of insulin resistance, dyslipidemia, and hypertension [2], leading to type 2 diabetes mellitus (T2DM) and enhanced risk of cardiovascular disease during adulthood [1]. Several investigations have confirmed that intensive lifestyle interventions can increase weight-loss as well as reduce the later risk of developing T2DM in children with obesity [3]. Nevertheless, lifestyle changes alone are not always effective [4]. On the other hand, there are no approved weight-loss medications for children under 12 years of age [4]. Metformin is the first-line oral anti-hyperglycemic agent approved by the US Food Drug Administration to treat T2DM in adults and children aged >10 years. Beyond its antidiabetic effects, metformin has been considered a promising compound for the amelioration of adolescent and childhood obesity; especially through the reduction of body mass index (BMI) Z-score and waist circumference (WC) [4,5,6]. 

According to the literature, there is considerable inter-individual variability in response to metformin. In relation to the glycemic response, although an important heritable component has been described (20–34%), there is only a few available genome-wide association studies (GWAS) and yet no consistently replicated genetic variants [7,8]. Fewer efforts have been made in the context of the anti-obesity action of metformin in children, with only two available pharmacogenetic studies [9,10]. The first one, which is focused on the metformin organic cation transporter 1 (OCT1) (*SLC22A1* gene), yielded controversial findings and reported the need for additional obesity targets to be analyzed in future approaches [9]. The other study is a pharmacokinetic approach in children with obesity, and could not identify any influence of genetic variants from the *OCT1* and *multidrug and toxin extrusion protein 1 (MATE1)* transporters on the pharmacokinetics of metformin [10].

GWAS and other genetic studies for BMI, waist-to-hip ratio, and other adiposity measures have identified more than 300 single-nucleotide polymorphisms (SNPs) that are strongly associated with obesity risk [11]. Interestingly, a pharmacogenetic approach focusing on these and other candidate genes might shed light on the action mechanism of metformin as a weight-reduction drug in children. At the same time, it might identify genetic variants that may be useful clinically to predict metformin efficacy.

On a previous work, we conducted a randomized control trial (RCT) in children with obesity and demonstrated that a six-month intervention with metformin decreases the BMI Z-score and improves inflammatory and cardiovascular-related obesity parameters [5]. Here, we conduct a genotyping study comprising hundreds of obesity and metformin candidate genes in 124 children, which are part of our previous RCT, with the aim to test whether common variants can predict the response to metformin intervention in terms of the post-treatment change in glucose metabolism, anthropometry, lipid metabolism, adipokines, and inflammatory markers. To our knowledge, this is the first candidate-gene pharmacogenetic approach focused on the effects of metformin in children with obesity. Pharmacogenetic studies such as this are crucial to provide new insight into the mechanisms regulating metabolic dysfunction and may point the way toward novel therapeutic targets for more precise interventions in childhood obesity.

## 2. Experimental Section

### 2.1. Study Design, Participants, and Intervention

The study was a multicenter and double-blind RCT, stratified according to sex and pubertal status in 160 children with obesity. Pubertal stage was determined according to Tanner criteria [12], and obesity was defined according to BMI by using the age and sex-specific cutoff points proposed by Cole et al. [13]. Children were randomly assigned to receive either (1 g/d) metformin or placebo for six months after meeting the defined inclusion criteria [14]. Figure S1 shows the flow diagram of participants throughout the study. All the details regarding study protocol, design, sample size, intervention, and participants (participant’s data collection and processing, samples codification, randomization method, double-blind condition, and adverse effects assessment) have been previously described [5,14]. The CONSORT statement (Consolidated Standards of Reporting Trials) has been considered in the study design report and the flow diagram (Figure S1).

### 2.2. Informed Consent and Ethics

All the patients and their parents/guardians were previously informed about the characteristics of the trial. The informed consent, read and signed, was mandatory to participate in this study. The study was conducted in accordance with the Declaration of Helsinki and received ethics approval. It was approved by the Ethics and Investigation Committees of the hospitals (Hospital Universitario Reina Sofía, Hospital Universitario de Santiago de Compostela, Hospital Clínico Universitario Lozano-Blesa, Hospital Universitario Virgen de las Nieves) at which the study was developed, whose reference was provided by the Ethics Committee for Biomedical Research of Andalusia on 15 January 2012 (acta 1/12) (ID code: 2010-2739). The study was registered by the European Clinical Trials Database (EudraCT, ID: 2010-023061-21) on 14 November 2011.

### 2.3. Blood Samples Collection

Blood samples were obtained between 08:30 and 10:30, and collected in overnight fasting conditions at the beginning and at the end of the trial, as previously reported [14]. For DNA extraction, peripheral white blood cells (buffy coat) were taken. All the samples were collected and stored frozen at −80 °C until analysis.

### 2.4. Anthropometric and Biochemical Measurements

Anthropometry, blood pressure, and serum concentrations of glucose, insulin, lipids (total cholesterol, triglycerides, high-density lipoprotein cholesterol (HDLc), low-density lipoprotein cholesterol (LDLc)), apolipoprotein A1 (Apo A1), and apolipoprotein B (Apo B) were measured, as previously reported [14]. The quantitative insulin sensitivity check index (QUICKI) and homeostasis model assessment for insulin resistance (HOMA-IR) were also calculated. Specific plasma adipokines, inflammation, and cardiovascular risk biomarkers (adiponectin, leptin, resistin, myeloperoxidase (MPO), total plasminogen activator inhibitor-1 (tPAI-1), tumor necrosis factor-alpha (TNF-α), interferon-γ (IFN-γ), C-reactive protein (CRP), monocyte chemoattractant protein-1 (MCP-1), interleukin-8 (IL-8), soluble intercellular adhesion molecule-1 (sICAM-1), and soluble vascular adhesion molecule-1 (sVCAM-1) were analyzed in duplicate by using XMap technology (Luminex Corporation, Austin, TX, USA) and human monoclonal antibodies (Milliplex Map Kit; Millipore, Billerica, MA, USA), as previously detailed [5].

Based on the adiponectin and leptin concentrations, the adiponectin–leptin ratio (ALR) was calculated.

### 2.5. DNA Extraction and Genotyping

The 140 individuals that completed the study intervention (68 treated children and 72 placebo) were included for the current genetic analyses. Genomic DNA was extracted from peripheral white blood cells using two kits, the Qiamp^®^ DNA Investigator Kit for coagulated samples and the Qiamp^®^ DNA Mini & Blood Mini Kit for non-coagulated samples (QIAgen Systems, Inc., Valencia, CA, USA). Genotyping analysis was performed by TaqMan allelic discrimination assay using the QuantStudio 12K Flex Real-Time PCR System (Thermo Fisher Scientific, Waltham, MA, USA).

#### Candidate Gene and SNP Selection

For the genotyping in the 140 DNA samples, we selected candidate genes and SNPs according to seven categories (Table S1): (1) SNPs in high-likelihood candidate genes for human obesity according to the literature and previous genotyping studies conducted by our research group; (2) SNPs in brown fat cell differentiation genes; (3) SNPs in differentially expressed genes according to a previous own microarray analysis of visceral adipose tissue in obese and normal-weight children; (4) SNPs identified by ongoing GWAS and big cohort studies for obesity and related metabolic traits in adult European populations; (5) SNPs related to metformin drug-metabolizing enzymes, transporters, and other previously reported pharmacogenetic targets; (6) SNPs predicted as binding sites of microRNAs related to obesity and metabolic dysfunction; and (7) SNPs in inflammation, oxidative stress, and antioxidant defense genes. During the SNP selection procedure, we used the Tagger program to capture (at r2 = 0.8) common (minor allele frequency (MAF) ≥5%) variants in European (CEU) HapMap population in these candidate genetic regions. The candidate genes and the number of SNPs analyzed per category are detailed in Table S1 [8,11,15,16,17,18,19,20,21,22,23,24,25,26,27]. Moreover, the genomic information for all the analyzed SNPs is summarized in Table S2. As a result, 255 SNPs on loci strongly associated with obesity as well as previously known metformin pharmacogenetic targets were finally genotyped. Our 255 selected markers map to 181 candidate loci across the human genome. 

**Figure 1:**
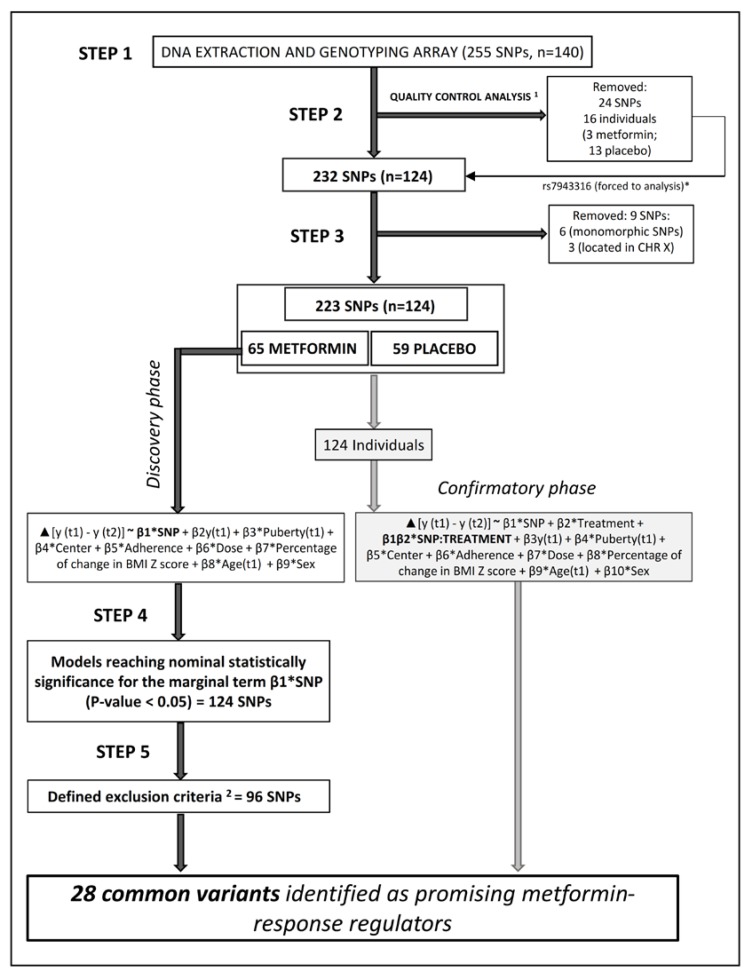
Workflow of the entire SNPs selection process for the statistical approach to identify the genetic variants as promising candidates of metformin-response regulation. ^1^ Quality control analysis, removed if: call rate per SNP <90%, call rate per subject <90%, HWE *p*-value < 0.05, MAF <5%, and LD is observed. * The SNP rs7943316 was forced to analysis and therefore integrated in the SNP selection process (more details in File S1). ^2^ Defined exclusion criteria: (a) SNPs presenting a weak *p*-value (defined as significant *p*-value ≥ 0.045) by trait analysis (removed = 11 SNPs); (b) SNPs only associated with one of the outcomes studied and not previously evidenced as metformin pharmacogenetic targets (removed = 57 SNPs); and (c) SNPs not showing a coherent behavior in their association across different phenotypes (regarding the direction of their beta estimates) and not previously evidenced as metformin pharmacogenetic targets (removed = 28 SNPs). Abbreviations: HWE, Hardy–Weinberg equilibrium; LD, linkage disequilibrium; MAF, minor allele frequency; SNP, single nucleotide polymorphism.

For the quality control analysis of all the candidate markers, we evaluated the linkage disequilibrium (LD), call rate, Hardy–Weinberg equilibrium (HWE), and MAF by experimental arm. In both the treatment and placebo arms, the MAFs of all SNPs were >5% and similar to those reported for Iberian populations in Spain in phase 3 of the 1000 Genomes Project. To account for the presence of genotyping errors, all SNPs and individuals with a <90% call rate were excluded from the analyses. In relation to HWE, Wigginton’s exact test [28] was applied at an alpha of 0.05, as in a cohort of 258 normal weight Spanish children [29]. After all quality control checks, 24 SNPs were removed. However, although due to significant deviation from HWE, the SNP rs7943316 should have been excluded, it was forced to analysis and therefore integrated in the SNP selection process (Figure 1; File S2). The reason was that it did not deviate from HWE in the Iberian population in Spain according to the frequency data presented from the 1000 Genomes Project in the *Ensembl* database (A|A: 0.112, A|T: 0.411 and T|T: 0.477). In addition, 16 individuals (three treated children and 13 placebo) were excluded due to a call rate <90%. This resulted in 232 markers from a final study population of 124 participants (65 treated children (32 boys) and 59 placebo (29 boys)) available for the statistical analysis. A complete workflow detailing the SNP selection procedure in the study population can be found in Figure 1. Additionally, all lists of SNPs excluded at each step of the selection process and more detailed information are available as a supplementary file (File S1).

Among them, there are genes strongly associated with several forms of obesity (including monogenic obesity), T2DM, as well as known drug targets or drug-metabolizing/transporting enzymes. A functional enrichment analysis (FEA) performed with the GeneTerm Linker R package revealed that they participate in important cellular processes and functions. Functional meta groups identified in the FEA analysis comprise: brown fat cell differentiation, cellular response to insulin stimulus, glucose homeostasis, regulation of blood pressure, response to oxidative stress, cellular component movement, response to glucocorticoid stimulus, protein kinase binding, cytokine-mediated signaling pathways, activation of adenylate cyclase activity, and respiratory electron transport chain.

Previously associated metformin pharmacogenetic variants that were not genotyped in our study comprise the *CAPN10*-rs3792269, the *OCT1*-rs628031, the *OCT1*-rs36056065, the *KCNQ1*-rs163184, and the *SP1*-rs784888. Thus, neither information nor new knowledge has been reported here for these variants.

### 2.6. Statistical Analysis

Pharmacogenetic analyses of metformin response were performed in the treatment arm as part of a discovery phase. However, since the application alone of a single-arm design could skip important pharmacogenetic behaviors (especially in the case of weight-loss interventions), a complementary phase to our treatment-arm approach was conducted including an SNP–treatment interaction term and placebo individuals (confirmatory phase). Especially for the case of weight-loss interventions, the inclusion of a secondary phase such as this is of special importance, helping in the confirmation of true pharmacogenetic regulators of metformin-induced weight-loss. That is to say, it will allow the final confirmation of genetic loci with effects seen in the treatment arm but not the control arm. Figure 1 also describes all the steps for the statistical analysis.

In both phases, we applied multiple linear regression models to test the effect of each SNP on metformin response under an additive genetic model of inheritage, where gi ϵ {0,1,2} is the number of minor alleles for the ith individual. Delta changes (T1–T2) for each outcome were calculated and used as dependent variables in the analyses. To address potential confounding, we implemented a variable selection procedure. Some variables were included in the models based on previous findings [5] or expert knowledge, while other variables were selected based on the backwards selection approach and the Bayesian information criterion. Final employed models after covariate selection can be found in File S2. Therefore, the covariates included in all the models were: the corresponding outcome, pubertal stage (prepubertal/pubertal), exact age (years) (all of them at baseline (T1)), center of recruitment (Hospital Universitario Reina Sofía, Hospital Universitario de Santiago de Compostela, Hospital Clínico Universitario Lozano-Blesa, Hospital Universitario Virgen de las Nieves), adherence (((pills ingested − pills returned)/pills predicted) × 100), dose (mg metformin or placebo/kg body weight), and sex. Additionally, models for outcomes strongly correlated to BMI Z-score (glucose metabolism, blood pressure, lipid metabolism, fat mass, adipokines, inflammation, and cardiovascular risk biomarkers) were further adjusted by the percentage of BMI Z-score change as a confounder. Furthermore, height was also considered as another confounder for the blood pressure outcomes [30]. Continuous variables and calculated deltas were tested for normality using the Shapiro–Wilk test and transformed when necessary by means of the natural log or the rank-based inverse normal transformation. All regression models were evaluated by model control (investigating the linearity of effects on outcome(s), consistency with a normal distribution, and variance homogeneity). All residuals versus fitted, normal Q-Q, scale location, and residuals versus leverage plots are available upon request.

We quantified the statistical power of our approach to detect modest genetic effects (F2 = 0.30) according to an alpha value of 0.05, a sample size of 65 metformin-treated children, and up to nine independent variables.

Correction for multiple tests requires special attention in genetic association studies. Given the high number of markers and collected measures, we considered several parallel approaches to correct for multiple hypothesis testing based on the number of SNPs and outcomes examined. Specifically, we employed multiple-test correction based on the methods proposed by Holm (1979), Hommel (1988), and Benjamini and Yekutieli (2001). To estimate the expected proportion of type I errors among the rejected hypotheses, we further computed false discovery rates (FDRs) as in Benjamini and Hochberg [31]. Given the presence of LD, the FDR method is a proper approach that does not assume independence between markers. Here, none of our findings underwent strict statistical correction for multiple hypotheses testing by FDRs. In this regard, the novel findings reported here should be viewed as hypothesis generating.

## 3. Results

### 3.1. Identification of 28 Common Variants as Promising Metformin Pharmacogenetic Markers

Among all the models that reached nominal statistically significance for the pharmacogenetic associations in the discovery phase (step 4: 124 SNPs; Figure 1), we removed 96 SNPs according to the exclusion criteria defined in the legend of the Figure 1. Although we tried to parameterize the process (more details are given in File S1), expert knowledge and a strict scientific criterion were the cornerstones during the SNP selection. Hence, as special conditions to maintain important SNPs in the analysis, we established SNPs that were considered as metformin pharmacogenetic targets [8,24,26,32,33] as well as SNPs that had presented a high *p*-value in their associations, as analyzed in step 4. Altogether, the selected markers represented the set of 28 common variants (Figure 1) distributed in 22 genes and mainly represented by intronic-like SNPs. Beyond the discovery phase, all associations were further interrogated for confirmation including a SNP–treatment interaction term and placebo individuals in the models. 

For the 28 selected SNPs, information related to SNP-type (exonic, ncRNA-intronic, promoter, UTR3’-5’, and intergenic variants), chromosome number, HWE, and MAF by experimental arm is presented in Table S3. In relation to HWE, all associated SNPs, except for rs7943316, hold equilibrium according to Wigginton’s exact test.

For both intervention groups, the general and clinical characteristics of the 124 children at the baseline and post-treatment stages, as well as the details of the statistical analysis in relation to the differences at baseline and post-treatment between groups are reported in the Table S4. 

**Figure 2:**
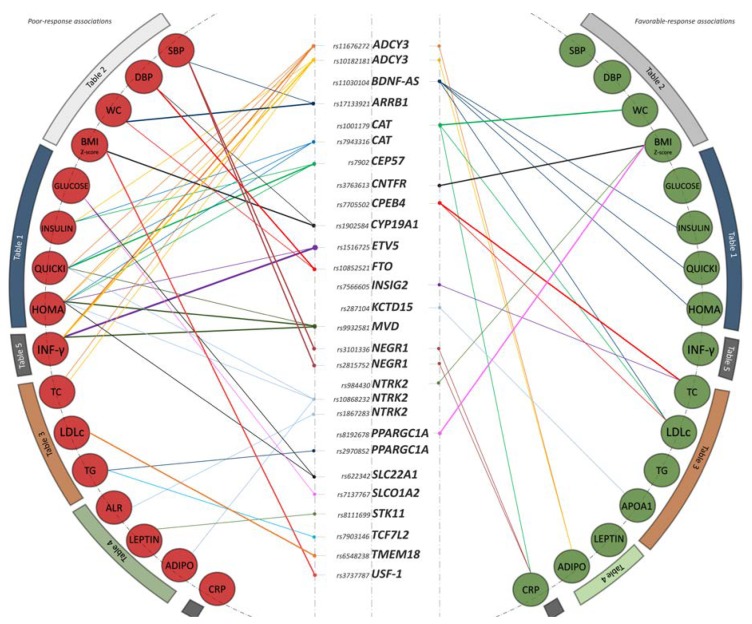
Interaction graph comprising all reported statistically significant associations in the discovery phase. Associations are clustered by phenotype block. The right half of the plot represents favorable-response associations, while the left half of the plot represents poor-response associations. Graph edges are weighted by the level of significance reported for each association. Abbreviations: ADIPO, adiponectin; ALR, adiponectin–leptin ratio; CRP, C-reactive protein; DBP, diastolic blood pressure; HOMA-IR, homeostasis model assessment for insulin resistance; INF-γ, interferon-γ; LDLc, low-density lipoproteins-cholesterol; QUICKI, quantitative insulin sensitivity check index; TC, total cholesterol; TG, triglycerides; SBP, systolic blood pressure; WC, waist circumference.

Among the reported pharmacogenetic associations, the results highlight a simultaneous effect of certain individual SNPs on several phenotypes as metformin-response regulators (Figure 2).

### 3.2. Glucose Metabolism

Most results and metformin pharmacogenetic targets were identified within the axis of glucose-related phenotypes, which includes fasting glucose, insulin levels, and the HOMA-IR and QUICKI indexes. Table 1 gathers all the results obtained for the 28 selected common variants in this phenotype block. Genetic variants in the loci *ADCY3*, *CAT*, *CEP57*, *ETV5*, *MVD*, *NTRK2*, *SLC01A2*, and *SLC22A1* behaved as poor-response markers after the six-month intervention (Figure 2). The most significant finding of the present block corresponded to the *CEP57*-rs7902 SNP and the QUICKI index change as outcome. This SNP stood out as a poor-response marker associated with a worsening in the ability of metformin to ameliorate the QUICKI index after the intervention (β = 0.49, confidence interval (CI) = (0.2, 0.78), *p*-value = 0.002). The result was consistent with a study-wide 62% FDR. Other poor-response associations were reported between the *MVD*-rs9932581, *SLC22A1*-rs622342, and *ADCY3*-rs11676272, and the HOMA-IR change as outcome (β = −0.45, CI = (−0.74, −0.16), *p*-value = 0.004; β = −0.38, CI = (−0.72, −0.04), *p*-value = 0.03 and β = −0.31, CI = (−0.58, −0.05), *p*-value = 0.03, respectively). On the contrary, the *BDNF-AS*-rs11030104 was the only variant underlined as a favorable pharmacogenetic marker in the block. Specifically, children carrying the G minor allele experienced an enhanced effect of metformin on fasting insulin levels (β = 0.48, CI = (0.11, 0.85), *p*-value = 0.01), HOMA-IR (β = 0.48, CI = (0.12, 0.83), *p*-value = 0.01), and QUICKI index (β = −0.47, CI = (−0.82, −0.11), *p*-value = 0.01) after the six-month intervention. All reported associations were independent of BMI Z-score.

### 3.3. Anthropometry and Blood Pressure

The results for anthropometry and blood pressure outcomes are presented in Table 2. Here, while genetic variants in the *ARRB1*, *CYP19A1*, *FTO*, *NEGR1* and *USF-1* genes behaved as poor-response markers, SNPs in the *CAT*, *CNTFR*, *NTRK2*, and *PPARGC1A* were reported as favorable pharmacogenetic targets (Figure 2). The top significant result of this phenotype block belonged to the *USF-1*-rs3737787 marker and the BMI Z-score change as outcome. The association implied a worsening in the response to metformin estimated in a less decrease, per the A allele copy, of BMI Z-score after the six-month intervention (β = −0.57, CI = (−0.91, −0.24), *p*-value = 0.001). The result was consistent with a study-wide 39% FDR. Similar direction in findings was obtained for the *FTO*-rs10852521 SNP and the outcomes WC and diastolic blood pressure (DBP) (β = −2.41, CI = (−4.44, −0.38), *p*-value = 0.02 and β = −4.79, CI = (−8.07, −1.52), *p*-value = 0.007, respectively). In relation to favorable-response pharmacogenetic markers, there was a remarkable association among the variants *PPARGC1A*-rs8192678, *CNTFR*-rs3763613, and *NTRK2*-rs984430, and the BMI Z-score change as outcome (β = 0.51, CI = (0.16, 0.87), *p*-value = 0.007; β = 0.55, CI = (0.19, 0.91), *p*-value = 0.004; and β = 0.56, CI = (0.05, 1.08), *p*-value = 0.03, respectively); as well as between the *CAT*-rs1001179 and the WC change (β = 3.03, CI = (1.24, 4.82), *p*-value = 0.002). All reported associations in blood pressure outcomes were independent of BMI Z-score and height. Further data obtained for weight and height outcomes did not show any significant association and consequently these are not presented here, but are available upon request.

### 3.4. Lipid Metabolism

Regarding lipid metabolism outcomes, variants in the *ADCY3*, *PPARGC1A*, *TCF7L2*, and *TMEM18* genes were associated with a worse response to metformin intervention (Table 3). Otherwise, variants in the *BDNF-AS*, *CAT*, *CPEB4*, *INSIG2*, and *KCTD15* were identified as favorable-response markers (Table 3 and Figure 2). The top findings of the present block corresponded to the SNP *TMEM18*-rs6548238, which was identified as a poor-response marker for the change in LDLc levels (β = −14.44, CI = (−23.88, −5), *p*-value = 0.005), and to the *CPEB4*-rs7705502, which was highlighted as a favorable-response marker for the change in total cholesterol levels (β = 13.81, CI = (4.77, 22.85), *p*-value = 0.005). These results were consistent with a study-wide FDR of 67% and 49%, respectively. The SNP *CPEB4*-rs7705502 was further identified as a favorable metformin-response marker for the change in LDLc levels. On the other hand, the SNPs *ADCY3*-rs11676272 and *ADCY3*-rs10182181 were again identified as poor-response pharmacogenetic targets, correlating with a worse ability of metformin to decrease total cholesterol levels in children carrying the effective A alleles (Table 3). In the same way, genetic variants in *TCF7L2* and *PPARGC1A* loci showed statistically significant results in relation to change in triglycerides levels. All the reported associations were independent of BMI Z-score. Data in relation to HDLc and Apo B did not show any significant association and consequently these are not presented here, but are available upon request.

### 3.5. Adipokines and Inflammatory Biomarkers

With regard to adipokines levels, we found the *STK11*-rs8111699 SNP as a poor-response marker for the change of leptin levels after the intervention (Table 4). Other poor-response associations of the block were reported between the *NTRK2* SNPs and the outcomes adiponectin and ALR changes. 

Finally, findings related to inflammatory biomarkers are presented in Table 5. Outstanding results from this block were reported for the INF-γ change as outcome and the poor-response markers *ETV5*-rs1516725 and *MVD*-rs9932581 (β = −1.13, CI = (−1.68, −0.59), *p*-value < 0.001 and β = −0.51, CI = (−0.82, −0.21), *p*-value = 0.002, respectively). These results were consistent with a study-wide 6% and 22% FDR respectively. Both SNPs also presented concordant associations as negative regulators of HOMA-IR metformin-response in previous blocks (Table 1 and Figure 2). Getting back to the INF-γ outcome, the *ADCY3*-rs11676272 and the *ADCY3*-rs10182181 were also underlined as poor-response markers. In this regard, children carrying the effective A alleles experienced a lower amelioration of their INF-γ levels after the intervention in comparison to major-allele carriers (β = −0.45, CI = (−0.73, −0.17), *p*-value = 0.003, and β = −0.45, CI = (−0.74, −0.16), *p*-value = 0.004, respectively). In relation to favorable-response markers, there was a remarkable association reported between the variant *CAT*-rs1001179 and the change in CRP levels (β = −0.49, CI = (0.05, 0.93), *p*-value = 0.03). All associations reported here were independent of BMI Z-score. No significant association was found for the following outcomes: resistin, MPO, tPAI-1, TNF-α, MCP-1, IL-8, sICAM-1 and sVCAM-1. Hence, they are not presented here, but are available upon request.

### 3.6. Confirmatory Phase

In order to confirm our findings, as we previously mentioned, all reported associations were evaluated as SNP–treatment interactions after the inclusion of placebo individuals in the analyses. As a result, up to 18, among all the previously described associations, remained statistically significant (marked with an asterisk in Table 1, Table 2, Table 3, Table 4 and Table 5). These associations can be understood as true pharmacogenetic phenomena and drug–gene interactions exclusive of the individuals belonging to the metformin arm.

## 4. Discussion

Our results show that the well-known variability in metformin response might have a genetic origin, also in the context of weight-loss and childhood obesity. We provide evidence for 28 common variants as promising pharmacogenetic regulators of metformin response in terms of a wide range of anthropometric and biochemical outcomes including glucose, lipid, and inflammatory traits (Figure 2). Our results not only support previously reported associations of variants in metformin transporters or targets (*SLC22A1*, *TCFL2* and *PPARGC1A*) but also identify novel and promising loci such as the *ADYC3* and the *BDNF-AS* genes, with biological relevance in the AMP kinase (AMPK) route and other metformin-related pathways. Despite the study initially being focused on the effect of metformin as an anti-obesity agent, the bulk of the findings and metformin pharmacogenetic targets were identified within the axis of glucose-related phenotypes (Figure 2). This, although striking, is to be expected, taking into account the well-reported glucose-lowering effect of metformin in T2DM European adult populations [8,24,26,32,33] and the improvement of insulin status in patients with hyperinsulinemia or insulin resistance [34,35]. In this regard, it might happen that some of the beneficial effects of metformin in childhood obesity could be mediated through an improvement of the impaired glucose metabolism. 

Among the novel and promising reported targets in our study, the *ADCY3* (adenylate cyclase 3) locus is especially interesting. Two SNPs within this gene were identified as poor metformin-response markers in the glucose, lipid, and inflammatory phenotype blocks (Figure 2). The ADCY3 protein is a member of the mammalian adenylyl cyclase family responsible for generating the second messenger cyclic adenosine monophosphate (cAMP) in human tissues. Several lines of evidence suggest the interesting possibility that the ADCY3 protein may play an important role in the regulation of adiposity as well as crucial physiological roles in mice muscle and liver [36], which are all target tissues of metformin. Likewise, it has been proposed that ADCY3 dysfunction in peripheral tissues could be related to metabolic disorders by inducing adipocyte dysfunction and insulin resistance in mice [36]. The molecular mechanism of this relation might underlie the dysregulation of the ATP/cAMP cellular balance and the resulting disruption of the PKA-induced AMPK activation. Taking it into account and given that AMPK is the main target by which metformin elicits its effects in the body, our *ADCY3* pharmacogenetic report merits special attention as a candidate gene for consideration in other genotyping and functional studies.

Other interesting findings involved two loci related to the brain-derived neurotrophic factor (BDNF) protein. These were the *BDNF-AS* region, which is an antisense RNA gene upstream the *BDNF*, and the *NTRK2* locus, which encodes the BDNF receptor protein. SNPs within these loci were robustly associated as favorable and poor-response markers respectively in all the analyzed phenotype blocks (Figure 2). The *BDNF-AS*–rs11030104 discovery is especially noticeable according to previous works indicating that the *BDNF-AS* intron region has a key role in regulating *BDNF* expression in humans [37]. Furthermore, our *BDNF-AS*–rs11030104 and other *BDNF* SNPs have been strongly associated with obesity risk [38] and weight response after intensive lifestyle modification [39]. BDNF is a neurotrophin that plays important functions in the central nervous system and systemic or peripheral inflammatory conditions such as acute coronary syndrome and T2DM. Interestingly, BDNF has been demonstrated to have strong anti-hyperglycemic and anti-inflammatory effects against the progression of T2DM [40]. Some studies have also revealed a strong effect of metformin as a *BDNF*-expression enhancer in mice [41,42]. On this matter, a recent review suggested that the correlation between BDNF and metformin might be the reason for metformin-induced insulin action by insulin receptor binding, metformin-induced high BDNF levels due to increasing AMPK, and enhanced tyrosine kinase receptor activity, which may amplify BDNF signaling [43]. Altogether, these findings suggest that the BDNF product could be a key element for the successful action of the drug against both obesity and T2DM conditions. Therefore, the *BDNF-AS*, *NTRK2*, and the *BDNF* locus could be good candidate pharmacogenetic targets to be studied in future human and *in vitro* studies.

On the other hand, we also provide evidence for exclusive and robust pharmacogenetic associations within anthropometric traits (Table 2 and Figure 2). Top findings within the block involved well-known obesity genes such as the *FTO*, the *CYP19A1* and the *USF-1*. Similar results for SNPs in the *FTO* gene have been reported in a previous metformin pharmacogenetic study for the BMI Z-score change in girls with androgen excess [44]. Given that *FTO* is a key obesity-associated gene and an important factor controlling feeding behavior and energy expenditure, it could be likely that metformin elicits direct actions on obesity via adiposity reduction. 

The most significant report in our study belonged to the poor-response marker *ETV5*-rs1516725 and the INF-γ change as outcome. With a study-wide FDR of 6%, this finding almost reached multiple testing correction significance. This genetic variant also presented concordant association as a negative regulator of the HOMA-IR response (Table 1 and Figure 2). According to literature, the *ETV5* gene has been associated with BMI in multiple GWAS studies [45,46] and functionally linked to obesity [47]. Specifically, ETV5 seems to have a critical role in regulating insulin secretion and glucose metabolism in mice, which might support our strong association as a metformin-response regulator [47]. Other novel genetic regions identified in our study comprised the loci *CEP57*, *CPEB4*, *CAT*, or the *SLC01A2*, which showed concordant associations across different phenotype blocks (Figure 2). Interestingly, the encoded proteins of these loci participate in molecular processes that are strongly related to the action mechanism of metformin via AMPK-independent pathways [48,49].

Regarding previously reported genes in the literature, our study identified some well-known pharmacokinetic and pharmacodynamic targets of metformin such as the metformin transporter *SLC22A1*-rs622342 and the transcription factors *TCFL2*-rs7903146 and *PPARGC1A* (rs2970852 and rs8192678), for which we here replicate all previously reported associations [8,50,51,52,53]. Other literature variants also announced in our study map within the loci *STK11*, *FTO*, *INSIG2*, and the *KCTD15*. For these variants, although we do not replicate exact results, we provide findings in line with those previously presented [44,54,55,56], thereby strengthening our proposal and broadening previous knowledge. 

We are aware of some limitations in the current study. 1) First, our observations are from a setting of multiple hypotheses testing, which only reach a nominal level of statistical significance. 2) Regarding null associations, there are variants such as the *SLC47A1*-rs2289669 or the *ATM*-rs11212617 which, in spite the wide backup of association in previous GWAS and candidate studies [24,33,50,57,58,59], have not reached nominally statistically significance in any of our analyses. Although the association of the *ATM*-rs11212617 as a pharmacogenetic marker remains controversial in the literature [25], the lack of significance for this and other markers in our study requires special attention. One reason for that could be a lack of statistical power in our design. Although we have reported enough statistical power 83.36% to detect previously described modest effects (F2 = 0.30), we actually have an inadequate power for detecting such small effect sizes such as those identified in GWAS studies. Notwithstanding, considering the number of variants likely to influence the phenotypes under study, even a submaximal power is likely to provide a number of true positive associations. On this matter, reported associations in the *ADCY3* locus and *BDNF*-related regions still merit consideration as true pharmacogenetic associations. 

## 5. Conclusions

In conclusion, we propose novel mechanisms by which genetics might contribute to variation in response to metformin as an anti-obesity agent in different traits. Both poor-responses and favorable-responses were identified, relying upon the allele copy to achieve an effect of metformin on glucose levels and insulin sensitivity, anthropometric parameters, blood pressure, lipid profile, adipokines, and inflammatory biomarkers. Genetic variants in promising loci such as the *ADYC3* and the *BDNF-AS* could explain the inter-individual variability in metformin response, and therefore clinically predict the metformin efficacy based on genetics. Although interesting, none of the reported associations remained statistically significant after multiple-test correction, and thus should be interpreted with caution. Certainly, these and other generated hypotheses require more detailed characterization in bigger and independent samples. Pharmacogenetic approaches such as this might provide new insight into mechanisms regulating metabolic dysfunction and may point the way toward novel therapeutic targets for more precise interventions in childhood obesity. 

## Figures and Tables

**Table 1:** Summary of association data for the 28 selected common variants in glucose metabolism outcomes.

			Δ Fasting Glucose		Δ Fasting Insulin		Δ HOMA-IR		Δ QUICKI	
SNP	Nearest Gene	Effect (Other) Allele	Β (95%CI)	*p*-Value	Β (95%CI)	*p*-Value	Β (95%CI)	*p*-Value	Β (95%CI)	*p*-Value
*rs11676272*	*ADCY3*	A (G)	0.08 (−2.57, 2.74)	0.95	−0.36 (−0.63, −0.08)	**0.01**	−0.31 (−0.58, −0.05)	**0.03**	0.31 (0.04, 0.59)	0.03
*rs10182181*	*ADCY3*	A (G)	−0.32 (−3.03, 2.39)	0.82	−0.34 (−0.62, −0.06)	**0.02**	−0.31 (−0.58, −0.04)	**0.03**	0.27 (−0.01, 0.55)	0.07
*rs17133921*	*ARRB1*	A (G)	0.70 (−4.32, 5.71)	0.79	0.33 (−0.2, 0.86)	0.23	0.32 (−0.18, 0.83)	0.22	−0.02 (−0.54, 0.51)	0.95
*rs11030104*	*BDNF-AS*	G (A)	2.43 (−1.08, 5.95)	0.18	0.48 (0.11, 0.85)	0.01	0.48 (0.12, 0.83)	**0.01**	−0.47 (−0.82, −0.11)	**0.01**
*rs1001179*	*CAT*	T (C)	0.43 (−2.89, 3.75)	0.80	0.27 (−0.1, 0.64)	0.16	0.24 (−0.12, 0.59)	0.20	−0.29 (−0.65, 0.06)	0.11
*rs7943316*	*CAT*	A (T)	−1.57 (−5.94, 2.8)	0.49	−0.63 (−1.07, −0.19)	**0.009**	−0.59 (−1.05, −0.13)	**0.02**	0.46 (0.04, 0.88)	**0.04**
*rs7902*	*CEP57*	A (G)	−1.02 (−3.82, 1.78)	0.49	−0.37 (−0.7, −0.05)	0.03*	−0.38 (−0.69, −0.08)	0.02*	0.49 (0.2, 0.78)	0.002*
*rs3763613*	*CNTFR*	T (G)	−0.78 (−4.35, 2.8)	0.67	0.17 (−0.2, 0.55)	0.36	0.15 (−0.2, 0.51)	0.41	−0.06 (−0.42, 0.3)	0.73
*rs7705502*	*CPEB4*	A (G)	−0.7 (−4.85, 3.47)	0.75	0.08 (−0.38, 0.54)	0.74	0.02 (−0.42, 0.47)	0.92	−0.06 (−0.51, 0.39)	0.8
*rs1902584*	*CYP19A1*	T (A)	2.6 (−3.66, 8.85)	0.42	0.45 (−0.29, 1.18)	0.24	0.35 (−0.36, 1.06)	0.34	−0.66 (−1.35, 0.03)	0.07
*rs1516725*	*ETV5*	T (C)	−3.85 (−9.04, 1.35)	0.15	−0.58 (−1.24, 0.07)	0.09	−0.70 (−1.3, −0.1)	**0.03**	0.57 (−0.05, 1.19)	0.08
*rs10852521*	*FTO*	T (C)	−2.60 (−5.98, 0.78)	0.14	−0.25 (−0.61, 0.12)	0.19	−0.25 (−0.59, 0.1)	0.17	0.15 (−0.21, 0.52)	0.40
*rs7566605*	*INSIG2*	C (G)	−2.03 (−5.13, 1.08)	0.21	−0.11 (−0.45, 0.23)	0.53	−0.19 (−0.51, 0.14)	0.26	−0.02 (−0.35, 0.32)	0.93
*rs287104*	*KCTD15*	G (A)	0.69 (−2.11, 3.48)	0.64	−0.11 (−0.45, 0.23)	0.53	−0.17 (−0.46, 0.13)	0.28	0.10 (−0.2, 0.4)	0.53
*rs9932581*	*MVD*	T (C)	−1.86 (−4.82, 1.1)	0.23	−0.43 (−0.75, −0.12)	**0.01**	−0.45 (−0.74, −0.16)	**0.004**	0.32 (0.01, 0.64)	0.05
*rs3101336*	*NEGR1*	T (C)	−0.90 (−3.85, 2.05)	0.55	−0.05 (−0.39, 0.28)	0.76	−0.08 (−0.4, 0.24)	0.64	−0.01 (−0.34, 0.31)	0.94
*rs2815752*	*NEGR1*	G (A)	−0.90 (−3.85, 2.05)	0.55	−0.05 (−0.39, 0.28)	0.76	−0.08 (−0.4, 0.24)	0.64	−0.01 (−0.34, 0.31)	0.94
*rs984430*	*NTRK2*	T (C)	1.88 (−2.71, 6.48)	0.43	0.06 (−0.46, 0.57)	0.83	0.16 (−0.33, 0.65)	0.52	0.10 (−0.38, 0.59)	0.67
*rs10868232*	*NTRK2*	G (A)	−2.51 (−6.78, 1.76)	0.26	−0.43 (−0.89, 0.02)	0.07	−0.52 (−0.94, −0.09)	**0.02**	0.49 (0.06, 0.93)	**0.03**
*rs1867283*	*NTRK2*	G (A)	0.84 (−2.08, 3.77)	0.57	−0.18 (−0.5, 0.13)	0.26	−0.18 (−0.49, 0.13)	0.26	0.07 (−0.24, 0.39)	0.64
*rs8192678*	*PPARGC1A*	T (C)	2.01 (−1.17, 5.19)	0.22	0.08 (−0.28, 0.44)	0.66	0.11 (−0.23, 0.45)	0.53	0.17 (−0.17, 0.52)	0.33
*rs2970852*	*PPARGC1A*	T (C)	1.01 (−1.92, 3.94)	0.50	−0.07 (−0.39, 0.26)	0.70	−0.05 (−0.36, 0.26)	0.76	0.01 (−0.3,0. 32)	0.95
*rs622342*	*SLC22A1*	C (A)	−3.70 (−6.95, −0.46)	**0.03**	−0.31 (−0.69, 0.06)	0.11	−0.38 (−0.72, −0.04)	0.03*	0.11 (−0.27, 0.49)	0.57
*rs7137767*	*SLCO1A2*	A (C)	−3.75 (−6.57, −0.93)	**0.01**	−0.06 (−0.43, 0.3)	0.74	−0.14 (−0.48, 0.2)	0.43	−0.05 (−0.41, 0.3)	0.77
*rs8111699*	*STK11*	C (G)	−0.65 (−3.71, 2.42)	0.68	0.09 (−0.26, 0.45)	0.62	0.12 (−0.22, 0.46)	0.48	−0.04 (−0.39, 0.3)	0.80
*rs7903146*	*TCF7L2*	T (C)	−1.93 (−5.1, 1.24)	0.24	0.18 (−0.18, 0.54)	0.33	0.05 (−0.24, 0.4)	0.77	−0.06 (−0.41, 0.29)	0.72
*rs6548238*	*TMEM18*	T (C)	−0.83 (−4.93, 3.27)	0.69	0.18 (−0.27, 0.64)	0.43	0.08 (−0.36, 0.51)	0.73	−0.16 (−0.6, 0.28)	0.47
*rs3737787*	*USF-1*	A (G)	0.37 (−2.84, 3.58)	0.82	0.25 (−0.12, 0.61)	0.19	0.22 (−0.12, 0.57)	0.21	−0.02 (−0.38, 0.34)	0.92

All analyses were adjusted for baseline age, sex, pubertal stage, center of recruitment, adherence to treatment, supplied dosage, and percentage of change in BMI Z-score (see Figure S1 for more details regarding employed regression models). Specific allele effects in the treatment arm are reported here (discovery phase). Listed *p*-values are not adjusted for multiple comparisons. Asterisks (*) indicate which associations reached statistically significance also as treatment–SNP interactions in the confirmatory phase. Abbreviations: B, beta; CI, confidence interval; HOMA-IR, homeostasis model assessment for insulin resistance; QUICKI, quantitative insulin sensitivity check index; SNP, single-nucleotide polymorphism.

**Table 2:** Summary of association data for the 28 selected common variants in anthropometry and blood pressure outcomes.

			Δ BMI Z Score		Δ WC (cm)		Δ DBP		Δ SBP	
SNP	Nearest Gene	Effect (other) Allele	Β (95%CI)	*p*-Value	Β (95%CI)	*p*-Value	Β (95%CI)	*p*-Value	Β (95%CI)	*p*-Value
*rs11676272*	*ADCY3*	A (G)	−0.27 (−0.58, 0.04)	0.09	−1.35 (−3.2, 0.5)	0.16	−2.58 (−5.39, 0.22)	0.08	−0.57 (−4.61, 3.47)	0.78
*rs10182181*	*ADCY3*	A (G)	−0.26 (−0.59, 0.07)	0.13	−1.43 (−3.31, 0.45)	0.14	−2.26 (−5.08, 0.56)	0.12	−1.13 (−5.29, 3.03)	0.60
*rs17133921*	*ARRB1*	A (G)	−0.14 (−0.76, 0.47)	0.65	−4.38 (−7.19, −1.56)	0.004*	−4.06 (−9.56, 1.44)	0.16	−8.22 (−15.58, −0.86)	0.03
*rs11030104*	*BDNF−AS*	G (A)	0.40 (−0.02, 0.81)	0.07	0.97 (−1.28, 3.22)	0.40	2.47 (−1.32, 6.26)	0.21	−2.59 (−8.1, 2.92)	0.36
*rs1001179*	*CAT*	T (C)	0.08 (−0.32, 0.49)	0.68	3.03 (1.24, 4.82)	0.002*	0.31 (−3.34, 3.96)	0.87	−1.59 (−6.51, 3.34)	0.53
*rs7943316*	*CAT*	A (T)	0.002 (−0.53, 0.54)	0.99	−1.92 (−4.58, 0.75)	0.17	0.69 (−4.12, 5.50)	0.78	1.68 (−4.66, 8.02)	0.61
*rs7902*	*CEP57*	A (G)	−0.21 (−0.57, 0.15)	0.26	1.07 (−0.52, 2.66)	0.19	0.79 (−2.43, 4.01)	0.63	−0.03 (−4.52, 4.46)	0.99
*rs3763613*	*CNTFR*	T (G)	0.55 (0.19, 0.91)	0.004*	0.87 (−1.17, 2.91)	0.41	0.27 (−3.31, 3.84)	0.88	2.27 (−2.81, 7.36)	0.39
*rs7705502*	*CPEB4*	A (G)	0.25 (−0.82, 0.26)	0.32	2.87 (0.02, 5.71)	0.05	−2.92 (−7.31, 1.47)	0.20	−2.62 (−8.99, 3.75)	0.43
*rs1902584*	*CYP19A1*	T (A)	−0.96 (−1.59, −0.32)	0.005*	1.63 (−2.14, 5.4)	0.40	−8.93 (−15.2, −2.67)	**0.008**	−5.67 (−15.70, 4.36)	0.27
*rs1516725*	*ETV5*	T (C)	0.54 (−0.05, 1.14)	0.08	0.52 (−2.51, 3.55)	0.74	−4.48 (−9.70, 0.73)	0.10	−8.49 (−15.99, −0.99)	0.03
*rs10852521*	*FTO*	T (C)	−0.27 (−0.67, 0.11)	0.17	−2.41 (−4.44, −0.38)	**0.02**	−4.79 (−8.07, −1.52)	**0.007**	−0.08 (−5.2, 5.04)	0.98
*rs7566605*	*INSIG2*	C (G)	−0.08 (−0.47, 0.32)	0.71	−0.30 (−2.3, 1.7)	0.77	−1.05 (−4.51, 2.42)	0.56	−1.72 (−6.63, 3.20)	0.50
*rs287104*	*KCTD15*	G (A)	−0.01 (−0.36, 0.35)	0.97	0.8 (−0.9, 2.5)	0.36	1.56 (−1.38, 4.51)	0.30	0.29 (−3.90, 4.47)	0.89
*rs9932581*	*MVD*	T (C)	0.12 (−0.22, 0.47)	0.49	−1.52 (−3.1, 0.06)	0.07	−0.64 (−4.01, 2.73)	0.71	−0.52 (−5.18, 4.15)	0.83
*rs3101336*	*NEGR1*	T (C)	−0.07 (−0.44, 0.3)	0.72	0.33 (−1.51, 2.16)	0.73	−2.51 (−5.55, 0.53)	0.11	−6.22 (−10.28, −2.15)	**0.005**
*rs2815752*	*NEGR1*	G (A)	−0.07 (−0.44, 0.3)	0.72	0.33 (−1.51, 2.16)	0.73	−2.51 (−5.55, 0.53)	0.11	−6.22 (−10.28, −2.15)	**0.005**
*rs984430*	*NTRK2*	T (C)	0.56 (0.05, 1.08)	0.03*	0.28 (−2.57, 3.12)	0.85	−0.67 (−5.67, 4.33)	0.80	−0.93 (−8.16, 6.29)	0.80
*rs10868232*	*NTRK2*	G (A)	0.10 (−0.4, 0.61)	0.69	0.36 (−2.25, 2.96)	0.79	−3.73 (−8.38, 0.93)	0.12	0.16 (−7.36, 7.68)	0.97
*rs1867283*	*NTRK2*	G (A)	−0.05 (−0.39, 0.3)	0.78	0.80 (−0.95, 2.55)	0.38	−1.79 (−4.89, 1.31)	0.27	−2.38 (−6.87, 2.10)	0.30
*rs8192678*	*PPARGC1A*	T (C)	0.51 (0.16, 0.87)	0.007*	−0.02 (−2.07, 2.04)	0.99	−0.26 (−3.76, 3.25)	0.89	1.57 (−3.73, 6.86)	0.57
*rs2970852*	*PPARGC1A*	T (C)	−0.33 (−0.68, 0.01)	0.06	−0.20 (−1.98, 1.59)	0.83	−0.90 (−4.02, 2.22)	0.57	−1.43 (−5.99, 3.12)	0.54
*rs622342*	*SLC22A1*	C (A)	0.14 (−0.25, 0.54)	0.47	2.14 (0.16, 4.12)	0.05	1.05 (−2.53, 4.63)	0.57	−0.57 (−5.44, 4.30)	0.82
*rs7137767*	*SLCO1A2*	A (C)	0.15 (−0.22, 0.52)	0.44	−0.44 (−2.46, 1.58)	0.67	0.19 (−3.18, 3.56)	0.91	−4.21 (−8.74, 0.32)	0.08
*rs8111699*	*STK11*	C (G)	−0.26 (−0.62, 0.1)	0.17	−1.27 (−2.94, 0.4)	0.14	−0.94 (−4.24, 2.35)	0.58	0.36 (−4.37, 5.09)	0.88
*rs7903146*	*TCF7L2*	T (C)	0.24 (−0.14, 0.62)	0.22	−0.15 (−2.17, 1.86)	0.88	−1.20 (−4.68, 2.28)	0.50	−1.71 (−6.68, 3.26)	0.50
*rs6548238*	*TMEM18*	T (C)	−0.43 (−0.93, 0.06)	0.09	1.57 (−0.9, 4.04)	0.22	1.86 (−2.47, 6.18)	0.41	0.40 (−5.84, 6.65)	0.90
*rs3737787*	*USF−1*	A (G)	−0.57 (−0.91, −0.24)	0.001*	−0.1 (−2.03, 1.83)	0.92	−2.80 (−6.11, 0.51)	0.11	−3.59 (−8.36, 1.18)	0.15

All analyses were adjusted for baseline age, sex, pubertal stage, center of recruitment, adherence to treatment, and supplied dosage. Additionally, the percentage of change in BMI and height for blood pressure outcomes (see Figure S1 for more details). Specific allele effects in the treatment arm are reported here (discovery phase). Listed *p*-values are not adjusted for multiple comparisons. Asterisks (*) indicate which associations reached statistically significance also as treatment–SNP interactions in the confirmatory phase. Abbreviations: B, beta; BMI, body mass index; CI, confidence interval; DBP, diastolic blood pressure; SBP, systolic blood pressure; SNP, single-nucleotide polymorphism; WC, waist circumference.

**Table 3:** Summary of association data for the 28 selected common variants in lipid metabolism outcomes.

			Δ LDLc		Δ Total Cholesterol		Δ Triglycerides		Δ Apo A1	
SNP	Nearest Gene	Effect (other) Allele	Β (95%CI)	*p-*Value	Β (95%CI)	*p-*Value	Β (95%CI)	*p-*Value	Β (95%CI)	*p-*Value
*rs11676272*	*ADCY3*	A (G)	−5.77 (−12.17, 0.63)	0.08	−8.57 (−14.91, −2.22)	0.01*	0.09 (−0.25, 0.42)	0.61	2.28 (−6.84, 11.4)	0.63
*rs10182181*	*ADCY3*	A (G)	−5.77 (−12.4, 0.85)	0.09	−8.77 (−15.03, −2.51)	0.009*	0.09 (−0.24, 0.42)	0.61	−0.44 (−9.48, 8.59)	0.92
*rs17133921*	*ARRB1*	A (G)	−4.93 (−16.57, 6.71)	0.41	−7.44 (−18.77, 3.89)	0.20	−0.25 (−0.82, 0.32)	0.40	2.33 (−11.17, 15.83)	0.74
*rs11030104*	*BDNF−AS*	G (A)	9.47 (1.07, 17.87)	**0.03**	8.38 (0.12, 16.63)	0.05	−0.38 (−0.8, 0.04)	0.09	0.58 (−11.45, 12.62)	0.92
*rs1001179*	*CAT*	T (C)	9.99 (2.34, 17.64)	**0.01**	7.73 (−0.13, 15.6)	0.06	−0.16 (−0.6, 0.29)	0.49	−5.44 (−16.15, 5.27)	0.33
*rs7943316*	*CAT*	A (T)	−0.98 (−11.82, 9.86)	0.86	1.02 (−9.48, 11.51)	0.85	0.12 (−0.41, 0.65)	0.66	2.35 (−10.96, 15.66)	0.73
*rs7902*	*CEP57*	A (G)	2.74 (−3.99, 9.47)	0.43	−0.62 (−7.37, 6.12)	0.86	0.13 (−0.21, 0.47)	0.47	−0.59 (−10.2, 9.01)	0.90
*rs3763613*	*CNTFR*	T (G)	2.50 (−5.86, 10.86)	0.56	3.33 (−4.8, 11.47)	0.43	−0.13 (−0.53, 0.27)	0.52	−3.52 (−14.83, 7.79)	0.55
*rs7705502*	*CPEB4*	A (G)	12.87 (3.31, 22.43)	**0.01**	13.81 (4.77, 22.85)	**0.005**	0.30 (−0.19, 0.79)	0.23	6.05 (−6.82, 18.91)	0.36
*rs1902584*	*CYP19A1*	T (A)	−1.27 (−16.78, 14.23)	0.87	0.62 (−15.15, 16.4)	0.94	−0.37 (−1.12, 0.37)	0.33	−14.67 (−37.91, 8.56)	0.23
*rs1516725*	*ETV5*	T (C)	7.95 (−4.99, 20.9)	0.24	1.54 (−10.95, 14.02)	0.81	0.25 (−0.32, 0.82)	0.39	−5.14 (−21.37, 11.08)	0.54
*rs10852521*	*FTO*	T (C)	5.27 (−2.78, 13.33)	0.21	3.68 (−4.31, 11.66)	0.37	−0.10 (−0.49, 0.29)	0.63	−4.86 (−15.38, 5.65)	0.37
*rs7566605*	*INSIG2*	C (G)	5.19 (−2.3, 12.67)	0.18	7.74 (0.67, 14.82)	**0.03**	0.26 (−0.1, 0.63)	0.17	4.71 (−6.65, 16.07)	0.42
*rs287104*	*KCTD15*	G (A)	−3.35 (−10.17, 3.46)	0.34	−4.06 (−11.22, 3.09)	0.27	0.08 (−0.26, 0.42)	0.65	−9.81 (−17.56, −2.06)	**0.02**
*rs9932581*	*MVD*	T (C)	−5.45 (−12.19, 1.28)	0.12	−5.45 (−12.45, 1.54)	0.13	0.12 (−0.25, 0.49)	0.52	−1.70 (−9.6, 6.2)	0.67
*rs3101336*	*NEGR1*	T (C)	2.22 (−5.03, 9.47)	0.55	1.45 (−5.63, 8.53)	0.69	−0.34 (−0.68, 0)	0.06	−2.85 (−12.17, 6.47)	0.55
*rs2815752*	*NEGR1*	G (A)	2.22 (−5.03, 9.47)	0.55	1.45 (−5.63, 8.53)	0.69	−0.34 (−0.68, 0)	0.06	−2.85 (−12.17, 6.47)	0.55
*rs984430*	*NTRK2*	T (C)	2.61 (−9.08, 14.3)	0.66	3.52 (−7.69, 14.73)	0.54	−0.03 (−0.58, 0.52)	0.91	5.15 (−8.86, 19.15)	0.48
*rs10868232*	*NTRK2*	G (A)	−0.77 (−11.43, 9.89)	0.89	2.05 (−8.37, 12.47)	0.70	0.08 (−0.45, 0.62)	0.76	11.95 (−0.08, 23.98)	0.06
*rs1867283*	*NTRK2*	G (A)	−0.47 (−8.03, 7.08)	0.90	−0.94 (−8.15, 6.27)	0.80	−0.10 (−0.45, 0.26)	0.59	−2.87 (−11.91, 6.17)	0.54
*rs8192678*	*PPARGC1A*	T (C)	2.96 (−5.14, 11.06)	0.48	4.43 (−3.32, 12.18)	0.27	0.06 (−0.33, 0.45)	0.77	4.69 (−5.55, 14.93)	0.38
*rs2970852*	*PPARGC1A*	T (C)	−0.60 (−8.01, 6.81)	0.87	−1.57 (−8.77, 5.64)	0.67	−0.36 (−0.7, −0.03)	**0.04**	1.80 (−7.26, 10.85)	0.70
*rs622342*	*SLC22A1*	C (A)	−0.99 (−8.87, 6.89)	0.81	−1.27 (−9.3, 6.77)	0.76	0.36 (−0.04, 0.76)	0.08	−2.15 (−11.77, 7.47)	0.66
*rs7137767*	*SLCO1A2*	A (C)	3.45 (−4.22, 11.12)	0.39	1.34 (−6.13, 8.8)	0.73	0.23 (−0.15, 0.6)	0.24	−7.87 (−17.37, 1.62)	0.12
*rs8111699*	*STK11*	C (G)	−5.23 (−12.3, 1.83)	0.15	−4.07 (−11.02, 2.88)	0.26	0.02 (−0.35, 0.39)	0.93	6.43 (−2.94, 15.81)	0.19
*rs7903146*	*TCF7L2*	T (C)	2.98 (−5.0, 11.0)	0.47	1.49 (−6.74, 9.73)	0.72	−0.44 (−0.8, −0.07)	0.02*	−10.90 (−20.81, −1)	**0.04**
*rs6548238*	*TMEM18*	T (C)	−14.44 (−23.88, −5)	**0.005**	−9.01 (−18.81, 0.8)	0.08	−0.08 (−0.59, 0.43)	0.76	0.18 (−11.8, 12.16)	0.98
*rs3737787*	*USF−1*	A (G)	2.51 (−5.4, 10.42)	0.54	0.71 (−7.05, 8.47)	0.86	−0.07 (−0.46, 0.32)	0.73	−2.64 (−12.02, 6.75)	0.59

All analyses were adjusted for baseline age, sex, pubertal stage, center of recruitment, adherence to treatment, supplied dosage, and percentage of change in BMI Z-score (see supplementary Figure S1 for more details regarding employed regression models). Specific allele effects in the treatment arm are reported here (discovery phase). Listed *p*-values are not adjusted for multiple comparisons. Asterisks (*) indicate which associations reached statistically significance also as treatment–SNP interactions in the confirmatory phase. Abbreviations: B, beta; CI, confidence interval; LDLc, low-density lipoprotein cholesterol; SNP, single-nucleotide polymorphism.

**Table 4:** Summary of association data for the 28 selected common variants in relation to adipokines and fat mass levels.

			Δ Adiponectin		Δ Leptin		Δ ALR		Δ Fat Mass	
SNP	Nearest Gene	Effect (other) Allele	Β (95%CI)	*p*-Value	Β (95%CI)	*p*-Value	Β (95%CI)	*p*-Value	Β (95%CI)	*p*-Value
*rs11676272*	*ADCY3*	A (G)	−0.43 (−0.76, −0.10)	0.01*	−1.82 (−4.21, 0.57)	0.14	−0.12 (−0.47, 0.22)	0.50	−0.23 (−1.84, 1.38)	0.78
*rs10182181*	*ADCY3*	A (G)	−0.47 (−0.79, −0.14)	0.008*	−2.17 (−4.59, 0.25)	0.09	−0.14 (−0.50, 0.22)	0.44	−0.21 (−1.86, 1.43)	0.80
*rs17133921*	*ARRB1*	A (G)	0.20 (−0.42, 0.82)	0.53	−2.32 (−6.78, 2.15)	0.32	0.36 (−0.27, 0.98)	0.27	−0.39 (−3.2, 2.43)	0.79
*rs11030104*	*BDNF−AS*	G (A)	0.14 (−0.33, 0.62)	0.56	−0.62 (−3.95, 2.71)	0.72	0.10 (−0.36, 0.56)	0.68	−0.48 (−2.6, 1.64)	0.66
*rs1001179*	*CAT*	T (C)	−0.16 (−0.61, 0.30)	0.51	0.17 (−2.89, 3.23)	0.91	−0.23 (−0.66, 0.21)	0.31	1.40 (−0.62, 3.41)	0.18
*rs7943316*	*CAT*	A (T)	0.24 (−0.34, 0.83)	0.42	0.81 (−3.55, 5.17)	0.72	0.36 (−0.26, 0.97)	0.27	−0.69 (−3.27, 1.89)	0.60
*rs7902*	*CEP57*	A (G)	−0.08 (−0.45, 0.30)	0.69	0.25 (−2.22, 2.71)	0.85	−0.22 (−0.62, 0.17)	0.28	0.22 (−1.52, 1.96)	0.80
*rs3763613*	*CNTFR*	T (G)	−0.05 (−0.50, 0.40)	0.84	0.92 (−2.37, 4.21)	0.59	0.05 (−0.42, 0.53)	0.83	0.19 (−1.79, 2.17)	0.85
*rs7705502*	*CPEB4*	A (G)	−0.51 (−1.05, 0.04)	0.07	−0.09 (−4.09, 3.92)	0.97	−0.21 (−0.76, 0.34)	0.46	−0.13 (−2.65, 2.4)	0.92
*rs1902584*	*CYP19A1*	T (A)	−0.21 (−1.05, 0.62)	0.62	−0.75 (−6.75, 5.24)	0.81	−0.53 (−1.38, 0.31)	0.22	2.67 (−0.92, 6.27)	0.15
*rs1516725*	*ETV5*	T (C)	0.06 (−0.74, 0.87)	0.88	−2.14 (−7.03, 2.75)	0.40	−0.03 (−0.82, 0.77)	0.94	1.07 (−2.14, 4.29)	0.52
*rs10852521*	*FTO*	T (C)	−0.06 (−0.50, 0.38)	0.79	−2.26 (−5.24, 0.72)	0.14	0.09 (−0.37, 0.54)	0.72	−1.38 (−3.27, 0.52)	0.16
*rs7566605*	*INSIG2*	C (G)	−0.07 (−0.49, 0.35)	0.75	0.06 (−2.85, 2.96)	0.97	0.14 (−0.28, 0.56)	0.52	−0.03 (−1.86, 1.8)	0.97
*rs287104*	*KCTD15*	G (A)	0.17 (−0.20, 0.54)	0.36	0.46 (−2.17, 3.08)	0.74	0.22 (−0.14, 0.58)	0.24	0.62 (−0.59, 1.83)	0.32
*rs9932581*	*MVD*	T (C)	0.001 (−0.41, 0.40)	0.99	−2.08 (−4.60, 0.44)	0.11	0.10 (−0.27, 0.47)	0.59	0.50 (−1.22, 2.22)	0.57
*rs3101336*	*NEGR1*	T (C)	0.16 (−0.23, 0.55)	0.42	−1.98 (−4.66, 0.69)	0.15	−0.02 (−0.41, 0.36)	0.90	0.72 (−1, 2.45)	0.41
*rs2815752*	*NEGR1*	G (A)	0.16 (−0.23, 0.55)	0.42	−1.98 (−4.66, 0.69)	0.15	−0.02 (−0.41, 0.36)	0.90	0.72 (−1, 2.45)	0.41
*rs984430*	*NTRK2*	T (C)	−0.03 (−0.66, 0.61)	0.94	−4.09 (−8.31, 0.12)	0.06	0.12 (−0.47, 0.71)	0.70	−0.08 (−2.77, 2.61)	0.95
*rs10868232*	*NTRK2*	G (A)	0.60 (0.06, 1.13)	**0.03**	1.39 (−2.60, 5.37)	0.50	0.29 (−0.24, 0.82)	0.29	1.63 (−0.87, 4.14)	0.21
*rs1867283*	*NTRK2*	G (A)	0.33 (−0.04, 0.70)	0.09	0.44 (−2.26, 3.14)	0.75	0.43 (0.08, 0.77)	0.02*	0.52 (−1.19, 2.22)	0.56
*rs8192678*	*PPARGC1A*	T (C)	−0.05 (−0.51, 0.42)	0.85	2.87 (−0.07, 5.81)	0.06	−0.10 (−0.54, 0.34)	0.67	−0.15 (−2.05, 1.75)	0.88
*rs2970852*	*PPARGC1A*	T (C)	0.13 (−0.25, 0.52)	0.50	−0.36 (−3.16, 2.44)	0.80	0.11 (−0.25, 0.48)	0.54	1.04 (−0.65, 2.72)	0.23
*rs622342*	*SLC22A1*	C (A)	−0.17 (−0.64, 0.30)	0.49	0.77 (−2.18, 3.72)	0.61	0.03 (−0.39, 0.44)	0.90	−0.02 (−1.93, 1.89)	0.98
*rs7137767*	*SLCO1A2*	A (C)	0.20 (−0.23, 0.64)	0.37	0.12 (−2.90, 3.13)	0.94	0.07 (−0.37, 0.50)	0.77	0.56 (−1.17, 2.29)	0.53
*rs8111699*	*STK11*	C (G)	−0.30 (−0.69, 0.09)	0.14	−2.76 (−5.17, −0.34)	**0.03**	−0.16(−0.54, 0.23)	0.43	−0.09 (−1.87, 1.68)	0.92
*rs7903146*	*TCF7L2*	T (C)	0.16 (−0.26, 0.59)	0.46	−1.26 (−4.22, 1.71)	0.41	0.13 (−0.28, 0.55)	0.54	0.98 (−0.9, 2.85)	0.31
*rs6548238*	*TMEM18*	T (C)	0.03 (−0.53, 0.60)	0.91	0.57 (−3.30, 4.44)	0.77	−0.33 (−0.86, 0.21)	0.24	0.94 (−1.46, 3.35)	0.45
*rs3737787*	*USF−1*	A (G)	−0.25 (−0.71, 0.20)	0.28	0.15 (−3.09, 3.39)	0.93	−0.45 (−0.86, −0.04)	0.05	0.70 (−1.17, 2.58)	0.46

All analyses were adjusted for baseline age, sex, pubertal stage, center of recruitment, adherence to treatment, supplied dosage, and percentage of change in BMI Z-score (see Figure S1 for more details regarding employed regression models). Specific allele effects in the treatment arm are reported here (discovery phase). Listed *p*-values are not adjusted for multiple comparisons. Asterisks (*) indicate which associations reached statistically significance also as treatment–SNP interactions in the confirmatory phase. Abbreviations: ALR, adiponectin–leptin ratio; B, beta; CI, confidence interval; SNP, single-nucleotide polymorphism.

**Table 5:** Summary of association data for the 28 selected common variants in relation to inflammatory biomarkers.

			Δ INF−γ		Δ CRP	
SNP	Nearest Gene	Effect (other) Allele	Β (95%CI)	*p*-Value	Β (95%CI)	*p*-Value
*rs11676272*	*ADCY3*	A (G)	−0.45 (−0.73, −0.17)	**0.003**	−0.26 (−0.64, 0.12)	0.19
*rs10182181*	*ADCY3*	A (G)	−0.45 (−0.74, −0.16)	**0.004**	−0.18 (−0.57, 0.20)	0.36
*rs17133921*	*ARRB1*	A (G)	0.21 (−0.33, 0.75)	0.44	−0.46 (−1.23, 0.31)	0.24
*rs11030104*	*BDNF−AS*	G (A)	0.11 (−0.34, 0.56)	0.63	0.25 (−0.24, 0.75)	0.32
*rs1001179*	*CAT*	T (C)	0.12 (−0.26, 0.50)	0.54	0.49 (0.05, 0.93)	0.03*
*rs7943316*	*CAT*	A (T)	−0.13 (−0.63, 0.36)	0.61	0.12 (−0.51, 0.75)	0.72
*rs7902*	*CEP57*	A (G)	−0.13 (−0.46, 0.20)	0.45	0.07 (−0.34, 0.49)	0.72
*rs3763613*	*CNTFR*	T (G)	−0.08 (−0.50, 0.33)	0.69	0.23 (−0.26, 0.71)	0.37
*rs7705502*	*CPEB4*	A (G)	−0.24 (−0.73, 0.24)	0.33	0.05 (−0.56, 0.66)	0.87
*rs1902584*	*CYP19A1*	T (A)	−0.10 (−0.88, 0.69)	0.81	0.50 (−0.44, 1.43)	0.31
*rs1516725*	*ETV5*	T (C)	−1.13 (−1.68, −0.59)	**<0.001**	0.06 (−0.73, 0.84)	0.89
*rs10852521*	*FTO*	T (C)	−0.13 (−0.50, 0.24)	0.51	0.18 (−0.27, 0.64)	0.43
*rs7566605*	*INSIG2*	C (G)	−0.27 (−0.63, 0.08)	0.14	−0.31 (−0.73, 0.11)	0.16
*rs287104*	*KCTD15*	G (A)	−0.14 (−0.47, 0.20)	0.43	0.40 (0.02, 0.79)	0.05
*rs9932581*	*MVD*	T (C)	−0.51 (−0.82, −0.21)	**0.002**	−0.11 (−0.52, 0.30)	0.61
*rs3101336*	*NEGR1*	T (C)	−0.06 (−0.40, 0.28)	0.72	0.57 (0.15, 1.00)	**0.01**
*rs2815752*	*NEGR1*	G (A)	−0.06 (−0.40, 0.28)	0.72	0.57 (0.15, 1.00)	**0.01**
*rs984430*	*NTRK2*	T (C)	0.09 (−0.46, 0.64)	0.75	0.46 (−0.19, 1.11)	0.17
*rs10868232*	*NTRK2*	G (A)	−0.24 (−0.74, 0.26)	0.36	−0.10 (−0.71, 0.50)	0.75
*rs1867283*	*NTRK2*	G (A)	−0.01 (−0.36, 0.34)	0.96	0.24 (−0.18, 0.65)	0.27
*rs8192678*	*PPARGC1A*	T (C)	0.02 (−0.35, 0.40)	0.91	−0.09 (−0.54, 0.36)	0.69
*rs2970852*	*PPARGC1A*	T (C)	−0.05 (−0.39, 0.29)	0.77	0.02 (−0.38, 0.42)	0.92
*rs622342*	*SLC22A1*	C (A)	−0.27 (−0.68, 0.13)	0.20	0.12 (−0.35, 0.60)	0.62
*rs7137767*	*SLCO1A2*	A (C)	−0.18 (−0.54, 0.19)	0.35	0.08 (−0.36, 0.53)	0.71
*rs8111699*	*STK11*	C (G)	0.21 (−0.14, 0.55)	0.25	−0.11 (−0.53, 0.31)	0.61
*rs7903146*	*TCF7L2*	T (C)	0.06 (−0.32, 0.45)	0.75	0.16 (−0.31, 0.62)	0.51
*rs6548238*	*TMEM18*	T (C)	0.43 (−0.04, 0.89)	0.08	−0.10 (−0.74, 0.53)	0.75
*rs3737787*	*USF−1*	A (G)	0.14 (−0.26, 0.53)	0.50	−0.37 (−0.83, 0.09)	0.13

All analyses were adjusted for baseline age, sex, pubertal stage, center of recruitment, adherence to treatment, supplied dosage, and percentage of change in BMI Z-score (see Figure S1 for more details regarding employed regression models). Specific allele effects in the treatment arm are reported here (discovery phase). Listed *p*-values are not adjusted for multiple comparisons. Asterisks (*) indicate which associations reached statistically significance also as treatment–SNP interactions in the confirmatory phase. Abbreviations: B, beta; CI, confidence interval; CRP, C-reactive protein; INF-γ, interferon-γ; SNP, single-nucleotide polymorphism.

## Supplementary Materials

The following are available online at https://www.mdpi.com/2077-0383/8/9/1471/s1: Figure S1: Flow diagram of participants; File S1: Description of the steps for the SNPs selection; File S2: Final employed statistical models for discovery phase and confirmatory phase; Table S1: Candidate genes and number of SNPs analyzed per selection category. Table S2: Genomic information for all SNPs analyzed. Table S3: Quality control parameters and genomic information for the 28 associated common variants. Table S4: Clinical characteristics of the study population at baseline and post-treatment stages.
